# Large-Scale Patterns of Genetic Variation in a Female-Biased Dispersing Passerine: The Importance of Sex-Based Analyses

**DOI:** 10.1371/journal.pone.0098574

**Published:** 2014-06-02

**Authors:** Monica Guerrini, Clizia Gennai, Panicos Panayides, Alan Crabtree, Iñigo Zuberogoitia, Alex S. Copland, Olga Babushkina, Paolo M. Politi, Dimitri Giunchi, Filippo Barbanera

**Affiliations:** 1 Department of Biology, Zoology and Anthropology Unit, University of Pisa, Pisa, Italy; 2 Game & Fauna Service, Ministry of Interior, Nicosia, Cyprus; 3 BirdLife Cyprus, Nicosia, Cyprus; 4 Icarus Environmental Studies, Logroño, Spain; 5 BirdWatch Ireland, Banagher, Ireland; 6 Laboratory of Avian Ecology and Bird Protection, Saint Petersburg State University, Saint Petersburg, Russian Federation; 7 Orti-Bottagone Nature Reserve, World Wildlife Fund, Piombino, Italy; 8 Department of Biology, Ethology Unit, University of Pisa, Pisa, Italy; Institute of Biochemistry and Biology, Germany

## Abstract

Dispersal affects the distribution, dynamics and genetic structure of natural populations, and can be significantly different between sexes. However, literature records dealing with the dispersal of migratory birds are scarce, as migratory behaviour can notably complicate the study of dispersal. We used the barn swallow *Hirundo rustica* as model taxon to investigate patterns of genetic variability in males and in females of a migratory species showing sex-biased dispersal. We collected blood samples (*n* = 186) over the period 2006 to 2011 from adults (*H. r. rustica* subspecies) nesting in the same breeding site at either high (Ireland, Germany and Russia) or low (Spain, Italy and Cyprus) latitude across Europe. We amplified the Chromo Helicase DNA gene in all birds in order to warrant a sex-balanced sample size (92 males, 94 females). We investigated both uniparental (mitochondrial ND2 gene) and biparental (microsatellite DNA: 10 loci) genetic systems. The mtDNA provided evidence for demographic expansion yet no significant partition of the genetic variability was disclosed. Nevertheless, a comparatively distant Russian population investigated in another study, whose sequences were included in the present dataset, significantly diverged from all other ones. Different to previous studies, microsatellites highlighted remarkable genetic structure among the studied populations, and pointed to the occurrence of differences between male and female barn swallows. We produced evidence for non-random patterns of gene flow among barn swallow populations probably mediated by female natal dispersal, and we found significant variability in the philopatry of males of different populations. Our data emphasize the importance of taking into account the sex of sampled individuals in order to obtain reliable inferences on species characterized by different patterns of dispersal between males and females.

## Introduction

Distribution, dynamics and genetic structure of natural populations can be severely affected by dispersal [1]–[3], i.e. the movement of an organism from its birthplace to its first breeding site (natal dispersal) or from one breeding site to another (breeding dispersal) [4]–[5]. Dispersal may be significantly different between sexes, as has been well documented in birds and mammals [3], [4], [6]. The costs/benefits of such asymmetry usually depend on the life history and the mating system of a given species [7]. In particular, dispersal tends to be female-biased in birds and male-biased in mammals [4], [8], [9]. Given that dispersal can significantly affect gene flow among populations [10], the dispersing sex may appear as genetically less structured. Hence, accounting for sex is fundamental not only to find out potential differences in dispersal [10]–[11] but also to reliably infer the genetic structure of populations, as the latter could be driven mainly by the philopatric sex [12].

The estimate of the dispersal rate in natural populations is often incomplete because it usually requires direct methods with intensive, large-scale and long-term demographic studies [10], [11], [13]. However, recent advances in the genetic techniques allowed researchers to integrate field investigation with molecular DNA analysis. The combined use of markers with different way of inheritance (mitochondrial *versus* nuclear DNA) represented the most suitable approach to infer discrepancy between the dispersal pattern of males and females. Indeed, differences in the genetic picture drawn by mitochondrial and nuclear markers are expected when sex-biased dispersal occurs [11]. Studies focusing on the application of population genetic tools to infer sex-biased dispersal are well known for vertebrates, more frequently in birds and mammals (e.g.: eiders [14], rodents [15]) than in amphibians and reptiles (e.g.: frogs [16], turtles [17]). Nevertheless, as exhaustively discussed by Møller *et al.* [18], dispersal of migratory birds is poorly studied because routes can greatly complicate the interpretation of the genetic scenario [19].

The barn swallow *Hirundo rustica* is a polytypic passerine bird widely distributed throughout most of the northern hemisphere [20]. This species is extensively studied with reference to its morphology and behaviour. For instance, recent studies disclosed significant patterns of morphological differentiation among European populations in a few characters (ventral coloration, tail streamers) known to be under sexual selection [21]–[24]. As far as the migratory behaviour is concerned, European barn swallows can be divided in two main groups: one breeds in south-western Europe and winters in central and western Africa, the other breeds in northern Europe and winters in southern Africa [25]. Differences in morphological and behavioural traits notwithstanding, the occurrence of some degree of genetic differentiation among European *H. rustica* populations has never been proved by using either mitochondrial or microsatellite DNA markers [23], [24], [26]. However, although the barn swallow is a female-biased dispersal species (males are more philopatric than females, e.g. [27]), the sex of the investigated individuals has never been taken into account in any genetic study focusing on this taxon.

In this work we aim at: (i) analysing the genetic variability of European barn swallow populations over a wide sampling area by means of markers from both uniparental (mitochondrial DNA: mtDNA) and biparental (microsatellite DNA) genetic systems; (ii) testing the consequences of sex-biased dispersal on the population genetic structure by comparing patterns of variation in a well balanced sample of males and females. Overall, the barn swallow represents an excellent model among migratory species to investigate patterns of genetic variation in males and females. While lack of genetic structure is expected for the whole sample size, we predict the occurrence of different genetic pattern between male and female barn swallows [12], [16].

## Materials and Methods

### Ethics statement

The barn swallow is not an endangered species in all trapping areas of this study. Samples were obtained in the same place (six localities) in different years. Adults were trapped with mist-nets. Samples (one blood droplet) were collected by means of wing venipuncture (brachial/radial/ulnar vein). Birds were not sedated and did not suffer any injury: all of them were released 10 min after blood collection.

We report here below the coordinates of the sampling localities (see also Table S1) together with the information about the permits issued for each specific area: (1) Tullynisk, Offaly (Ireland: 53°07′N, 07°54′W). The licensing authority in Ireland is the National Parks and Wildlife Service (NPWS), which provided an annual (renewable) license for the present study (NPWS references 57/2009 and C41/2010). Separately, the capture of birds is also controlled through the British Trust for Ornithology Ringing Scheme. All licenses and permits were obtained by, and in the name of, A.S. Copland (BTO permit number A5115). All samples were taken from birds at a privately-owned site. Access to this site was arranged through the regional staff of the NPWS, who also have contact details for the owner/manager; (2) Itzehoe (Germany: 53°56′N, 09°31′E). Samples were collected by S. Martens, who is ringer at the Institute for Avian Research “Vogelwarte Helgoland”. Samples were collected at a private farm and future permissions should be requested to the owner of the same; (3) Lake Ladoga (Russian Federation: 60°40′N, 32°56′E). Samples were collected by O. Babushkina at the Ladoga Ornithological Station of the Biological Research Institute of the Saint Petersburg State University. The Ladoga Ornithological Station is comprised within the Nizhne-Svirsky State Reserve. All work at the station adhered to the current legislation of the Russian Federation and to the institutional guidelines of the State University of Saint Petersburg. No specific permits were needed to O. Babushkina. Future permissions should be requested to the Biological Research Institute of the Saint Petersburg State University; (4) Gorliz (Spain: 43°24′N, 02°57′W). Samples were collected by I. Zuberogoitia. He obtained a license to trap barn swallows and collect blood samples by the Department of Medio Ambiente (Diputación Foral de Bizkaia). The same administration manages the experimental farm for the selection of cow races where the sampling was carried out, and all permissions were obtained in the same site. Future permissions should be requested to the same Department; (5) Orti-Bottagone Nature Reserve (Italy: 42°57′N, 10°35′E). Samples were collected by R. Ceccherelli (veterinary, CRUMA, Leghorn). Land is a private property (oasis) of World Wildlife Fund (WWF) and future permissions should be requested to WWF Italy; (6) Polis (Cyprus: 35°02′N, 32°25′E). Samples were collected in a government land by A. Crabtree (Vice Chairman and Ringing Officer of BirdLife Cyprus), who obtained a specific permit from the competent authority of Cyprus (Game & Fauna Service, Ministry of Interior, Nicosia). Future permissions should be requested to the same Ministry.

### Biological sampling

**Figure 1:**
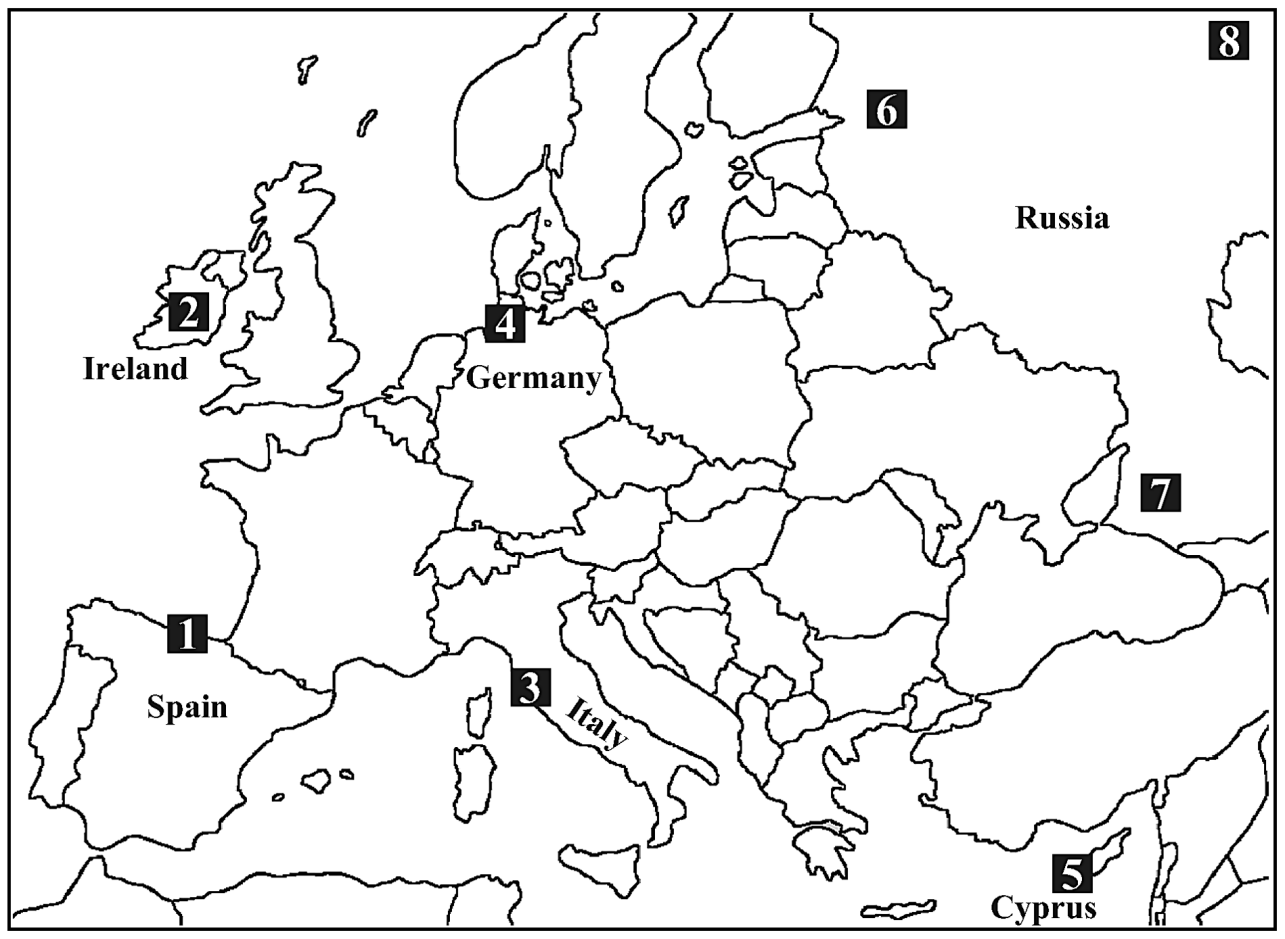
Barn swallow breeding populations sampled in this study. (1): SPA, Spain; (2): IRE, Ireland; (3): ITA, Italy; (4): GER, Germany; (5): CYP, Cyprus; (6): RUS, Russia. Mitochondrial DNA sequences available in the GenBank were obtained from Russian populations of Krasnodar (7, KRD) and Medvedevo (8, MED) (Table S1).

Blood samples (*n* = 186) were collected in subsequent years (2006–2011) from nesting adults (*H. r. rustica* subspecies) at the same breeding site in six areas across Europe (Figure 1, Table S1): Gorliz, Spain (SPA, *n* = 32); Tullynisk, Ireland (IRE, *n* = 26); Orti-Bottagone Nature Reserve, Italy (ITA, *n* = 33); Itzehoe, Germany (GER, *n* = 33); Polis, Cyprus (CYP, *n* = 32); Lake Ladoga, Russia (RUS, *n* = 30). One blood droplet was collected on filter paper (Whatman, UK) from each bird by wing venipuncture. Each swallow was ringed and sexed according to a PCR-based assay (see below).

### DNA extraction

Genomic DNA was extracted using the Puregene Core Kit-A (Qiagen, Germany) following the manufacturer's instructions. DNA content and purity were determined with an Eppendorf BioPhotometer (AG Eppendorf, Germany).

### Sexing

Chromo Helicase DNA (CHD) gene of ZZ (males) and ZW (females) sexual chromosomes was amplified with primers L1237 (5'-GAGAAACTGTGCAAAACA-3') and H1272 (5'-TCCAGAATATCTTCTGCTCC-3') previously tested in other bird species [28]. PCRs were prepared in 25 µL as in [29] and performed in a MyCycler thermal cycler (Biorad, USA) with the following profile: 3 min at 94°C, 30 cycles of 30 s at 94°C, 1 min at 48°C and 45 s at 72°C, finally 7 min at 72°C. PCR products were run in a 3.5% agarose gel for 60 min together with positive controls for male and female individuals. We set-up the PCR-based procedure by testing 20 barn swallows whose sex (10 males, 10 females) was determined through the inspection of standard morphological traits [30] by one of us (P.M. Politi).

### Mitochondrial DNA

#### Laboratory procedure

The entire mtDNA gene codifying for the second sub-unit of the NADH dehydrogenase (ND2, 1041 bp) was amplified using primers L5216 and H6313 [31]. PCRs (50 µL) were run in a MyCycler thermal cycler (Biorad) as in [29]. PCR products were purified using GenElute PCR Clean-up Kit (Sigma Aldrich, Italy) and directly sequenced on both DNA strands using the BigDye Terminator v. 3.1 Cycle Sequencing Kit on an ABI 3730 DNA automated sequencer (Applied Biosystems, USA) at Genechron (Rome, Italy). We amplified the ND2 gene in a subset of samples (10 for each population, *n* = 60), and we included in the alignment (clustalx v. 1.81: [32]) 16 sequences from the GenBank (Russia: Krasnodar Kray, KRD, *n* = 7 and Arkhangel'sk Oblast', MED, *n* = 9: [33]) (Figure 1, Table S1).

#### Population genetic inferences

We used dnasp v. 5.1 [34] to infer the mtDNA haplotypes. A network was constructed using dna alignment v. 1.3.3.1 (2003–2013 Fluxus Technology, UK) and the Median Joining method of [35] with network v. 4.5.1.0 (2004–2009 Fluxus Technology). We used arlequin v. 3.5.1 [36] to: (i) calculate the haplotype diversity (*h*), the nucleotide diversity (π) and the mean number of pairwise differences (*k*); (ii) investigate the partition of the mtDNA diversity (Analysis of the Molecular Variance, amova) among and within the populations using the Phi_ST_ analogous to Wright's *F*-statistics (1000 permutations) [37]; (iii) compute the average genetic distance among populations (1000 replicates with the TN93 algorithm) [38].

#### Historical demography

Inferences of historical demography were obtained using dnasp and different statistics as described in [39]. The analysis included (i) males and females plus the GenBank sequences (*n* = 60+16 = 76), (ii) only the males (*n* = 27, no GenBank entries), and (iii) only the females (*n* = 33, no GenBank entries). Ramirez-Soriano *et al*. [40] investigated the statistical power of a wide range of statistics computed on DNA polymorphism data in detecting a sudden population expansion, a sudden contraction or a bottleneck. They found that the most powerful tests were those based on haplotype frequencies, including the *F*_S_ of Fu [41] and the *R*_2_ statistic [42]. In this study, the significance of the *F*_S_ and *R*_2_ statistics was investigated by examining the null distribution of 5000 coalescence simulations using dnasp. Only significant negative *F*_S_ and positive *R*_2_ values were retained as evidence of population expansion [39]. We also computed the Tajima's *D* [43]. Nevertheless, [42] reported that *R*_2_ statistic has a greater power than the Tajima's *D* or *F*_S_ to detect population expansion when the sample size is small (∼10). Furthermore, the McDonald-Kreitman test [44] as implemented in dnasp was conducted for the entire dataset to investigate the deviation from an equal ratio of non-synonymous (K_a_) to synonymous (K_s_) fixed substitutions. Specifically for this test we used two US *H. r. erythrogaster* samples as outgroup (UWBM 78832 and UWBM 80547 from the University of Washington Burke Museum of Natural History, Seattle, USA; GenBank accession codes: HF548593-94).

The Mismatch Distribution (MD) of mtDNA pairwise differences was also examined using arlequin (males + females, males only, females only). The more ragged the shape of the distribution the closer was the population to a stationary model of constant size over a long period (raggedness index, *r*) [45]. The MD test uses the observed parameters of the expansion to perform coalescent simulations and to create new estimates of the same parameters. Departure from a model of sudden expansion was tested for each population by summing the squared differences (SSD) between observed and estimated MD [46], [47].

### Microsatellite DNA

#### Laboratory procedure

All samples (*n* = 186) were investigated at 10 loci of the microsatellite DNA (Short Tandem Repeats, STR) reported in [48], [49]. PCRs (12.5 µL) were performed as in [50] (Table 1). Gene sizing was carried out at the Research Centre of Clinical and Molecular Genetics (Pisa, Italy) on an ABI Prism 3730 DNA automated sequencer using genescan (Applied Biosystems). For the statistical analyses we used either the whole sample size (*n* = 186) or males (*n* = 92) and females (*n* = 94) separately.

**Table 1:** The characteristics of the investigated STR loci are shown.

Locus	*T*_M_ (°C)	Size-range (bp)	Repeat motif	A	*H_O_*	*H_E_*	*P*_ID_	*P*_ID_sib
Hir7	TD 52-50	215–273	(CT)_2_	25	0.69	0.94	7.07×10^−3^	2.82×10^−1^
Hir24	TD 56-54	194–238	(AGTG)_4_	12	0.46	0.88	2.06×10^−4^	9.06×10^−2^
Hir10	TD 52-50	147–201	(GTTT)_4_	13	0.75	0.85	8.79×10^−6^	3.06×10^−2^
Hru5	TD 52-50	110–140	(GT)_9_	15	0.66	0.84	4.01×10^−7^	1.05×10^−2^
Hir20	TD 50-48	231–275	(ATAG)_9_	15	0.76	0.84	1.90×10^−8^	3.61×10^−3^
Hir6	TD 56-52	178–218	(TCTA)_11_	11	0.80	0.84	9.19×10^−10^	1.24×10^−3^
Hir11	TD 50-48	166–214	(GATA)_7_	12	0.78	0.83	4.71×10^−11^	4.34×10^−4^
Hir4	TD 56-54	259–297	(GTTT)_5_	16	0.39	0.79	3.22×10^−12^	1.63×10^−4^
Hir5	TD 50-48	213–233	(GTTT)_4_	6	0.45	0.71	4.48×10^−13^	6.98×10^−5^
Hir15	TD 50-48	209–249	(ATGT)_2_	9	0.55	0.47	8.16×10^−14^	3.29×10^−5^

*T*_M_ (°C), annealing temperature; TD, touch-down PCR; A, number of alleles per locus; *H*_O_, mean observed heterozygosity; *H*_E_, mean expected heterozygosity; *P*_ID_, probability that two individuals drawn at random share identical genotypes; *P*_ID_sib, probability of identity among siblings. STR loci are sorted according to the increasing order of their *P*_ID_ (*P*_ID_sib) single-locus values (i.e., the locus at the top is the most informative one), and a sequentially multi-loci *P*_ID_ (*P*_ID_sib) is reported for each locus.

#### Genetic variability and relatedness

The discriminatory power of the whole set of STR loci was evaluated with gimlet v. 1.3.3 [51] by estimating the probability that two individuals drawn at random from the populations showed identical multilocus genotypes by chance (*P*_ID_ and *P*_ID_ sib: for the latter, we assumed sibling relationships) [52], [53]. Moreover, all loci were investigated using micro-checker v. 2.2.3 [54] to check for null alleles, allele dropout and scoring errors due to stuttering. Arlequin, fstat v. 2.9.3 [55] and genepop v. 3.4 [56] were used in order to: (i) compute the number of alleles per locus, the number of unique alleles and the allelic richness; (ii) calculate expected (*H*_E_) and observed (*H*_O_) heterozygosity; (iii) infer deviations from both Hardy-Weinberg Equilibrium (HWE) and Linkage Disequilibrium (LE) (10 000 dememorisations, 100 batches, 5000 iterations per batch); (iv) estimate gene flow (*N*_e_m, effective number of migrants per generation) via the private allele method of Slatkin [57]; (v) investigate the partition of the STR diversity within and among populations by amova; (vi) infer the degree of genetic differentiation among populations by estimating the average *F*_ST_ distance values. Bonferroni correction [58] was adopted to adjust the significance level of each test. The average *F*_ST_ distance values were plotted on the first two axes of a Principal Component Analysis (PCA) using statistica 5.0/W (Statsoft Inc., USA).

#### Population genetic structure

Bayesian clustering analysis was performed with structure v. 2.3.4 [59] to investigate the spatial structure of the genetic diversity. We focused on identifying the *K* (unknown) clusters of origin of the sampled individuals and to simultaneously assign them to each cluster. We assumed correlated allele frequencies and we used a prior population information option to take the sampling locality into account [60]. All simulations were run with 10^6^ Markov Chain of Monte-Carlo iterations, following a burn-in period of 10^5^ iterations, and were replicated five times per each *K*-value (1 to 12). The number of clusters that best fitted to the data was chosen using the formula of Evanno *et al*. [61]. An identification threshold to each cluster was selected (Q_i_ = 0.80) as in [62].

#### Sex-biased dispersal

In order to test for possible differences in the dispersal rate between males and females, fstat was used to calculate five different parameters: *F*_IS_, *F*_ST_, relatedness (*R*), mean (*mAI*_C_) and variance (*vAI*_C_) of the assignment index (*AI*_C_) within each sex [10]. The latter estimates the probability that a given genotype originates from the population where it was sampled, and the statistical significance is determined by a two-tailed test using 10 000 randomizations. Low *mAI*_C_ and high *vAI*_C_ values are interpreted for the dispersing sex. While *F*_ST_ and *R* are expected to be larger in the philopatric than in the dispersing sex, the opposite occurs for *F*_IS_. Nevertheless, the power of these statistics depends on dispersal rates, bias intensity, sampling design and the number of loci [10].

## Results

### Sexing

The CHD gene was amplified in all barn swallows: 92 birds were identified as males (single PCR product, ca. 200 bp) while 94 as females (two PCR products, ca. 200 and 240 bp).

### Mitochondrial DNA

#### Population genetics

**Figure 2:**
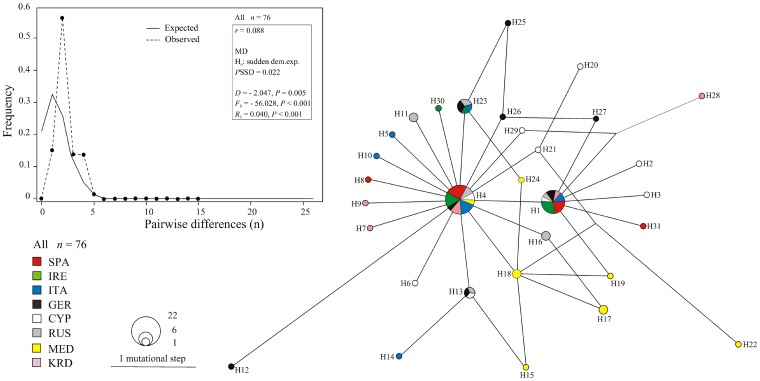
Median-Joining network of barn swallow populations computed on mtDNA haplotypes with network. Size of circles is proportional to the haplotype frequency. The colour of each population is indicated as well as the number of each haplotype. A length bar to compute the number of mutational changes was provided (see also Table S1). The inset showed Mismatch Distribution (MD) of the mtDNA pairwise differences (observed: dotted; expected: line) computed on the whole dataset (*n = *76). The expected curve was obtained from simulated values computed from the data under the model of demographic expansion (H_0_). The Harpending's raggedness index (*r*) was given with the *P* value of the SSD test as well as Tajima's *D*, Fu's *F*_S_ and *R*_2_ statistics.

The alignment of 76 (60+16) ND2 sequences produced 31 haplotypes (H1-H31: GenBank accession codes HF548562-HF548592) including 27 polymorphic sites. Estimates of all parameters are summarized in Table S2. SPA and IRL populations showed the lowest number of haplotypes as well as the lowest values of haplotype diversity (*h*), average number of pairwise differences (*k*) and nucleotide diversity (π). The highest number of haplotypes was found in CYP and MED. The Median Joining network showed that all barn swallow populations were genetically admixed with no noticeable divergence among haplotypes (Figure 2). In particular, two haplotypes (H1 and H4) were common to all populations. The 88.4% of the mtDNA variability was partitioned within populations while the 11.6% among them (Phi_ST_ = 0.131, *P*<0.001: data not shown). Only MED significantly diverged from all other populations (Phi_ST_ range: 0.28–0.38, all *P*<0.001, Table S3). When amova was performed either without KRD and MED populations or excluding MED only, we found that the partition of the mtDNA variability among populations decreased to 1.86% and 1.33%, respectively (Phi_ST_ = 0.02 and 0.01, respectively, *P*>0.05: data not shown). Finally, when all the estimated parameters were computed by including in the analysis males and females separately, all results matched those produced using the entire dataset (data not shown).

#### Historical demography

With the sole exception of the SSD test (*P* = 0.022), the statistics we used (*D* = −2.047, *F*_S_ = −56.028 and *R*_2_ = 0.040: all *P*<0.001) could not exclude the occurrence of a demographic expansion for the entire sample size (*n* = 76) (Figure 2). Indeed, the bell-shaped (*r* = 0.088) curve obtained for the Mismatch Distribution (MD) of the pairwise differences suggested a rapid population growth. When the demographic parameters were computed using males (*n* = 27) and females (*n* = 33) (GenBank entries excluded) separately, males showed exactly the same trend as the whole dataset (*D* = −1.970, *F*_S_ = −13.178 and *R*_2_ = 0.056: all *P*<0.001; SSD test: *r* = 0.158, *P* = 0.023), while for the females a demographic expansion was suggested by all statistics (*D* = −1.571, *F*_S_ = −18.449 and *R*_2_ = 0.064: all *P*<0.001; SSD test: *r* = 0.123, *P* = 0.063). The McDonald-Kreitman test was not significant for the whole sample size (Fisher exact test, *P* = 0.16).

### Microsatellite DNA: all individuals

#### Genetic variability

All STR loci employed were highly polymorphic. In the entire sample size (*n* = 186) the STR panel was powerful in discriminating individuals (*P*_ID_ = 8.16×10^−14^ and *P*_ID_sib = 3.29×10^−5^, Table 1), as values lower than 0.001 can be considered as satisfactory [53]. Micro-checker did not provide evidence for allele dropout or scoring errors due to stuttering, although three loci (Hir4, Hir7 and Hir24) showed an excess of homozygotes for most of the allele-size classes, thus pointing to the possible presence of null alleles (data not shown). The total number of alleles at each locus ranged between 6 and 25 (Hir5 and Hir7, respectively), with a mean of 13.4 alleles per locus (Table 1).

The average values of *H*_O_ were smaller than *H*_E_ for each locus (Fisher exact test, *P*<0.001 all loci, Table 1) and ranged between 0.39 and 0.80 (Hir4 and Hir6, respectively: Table 1). There was no evidence of LE at any pair of loci after sequential Bonferroni correction (*P*>0.05, all comparisons: data not shown). Both the number of alleles and gene diversity of each locus pointed to a very high degree of genetic variability. Allelic richness ranged between 7.9 and 9.9 (SPA and CYP, respectively: Table 2); CYP showed the highest number of private alleles (*n* = 8, Table 2). Average levels of *H*_O_ and *H*_E_ across all loci and populations were relatively homogeneous and showed very restricted ranges (*H*_O_: 0.60–0.67, *H*_E_: 0.79–0.82, Table 2). *H*_O_ values pointed to a deficiency of heterozygotes in all populations, which was confirmed by departure from HWE (*P*<0.001 all loci, Table 2). One locus in IRE, two loci in SPA, three loci in GER and RUS, and four loci in ITA and CYP were not in HWE (*P*<0.001, data not shown). However, we found that no locus deviated from HWE in all populations, and when the Bonferroni's correction was taken into account only three loci (Hir4, Hir7 and Hir24) deviated from HWE in some populations yet not in all (*P*<0.001, data not shown).

**Table 2:** The genetic variability of the STR loci for each population.

	Population	*n*	*n*_a_	*A*_r_	*A*_u_	*H*_O_	*H*_E_	*P*_HWE_	χ^2^ (*df*)	*F*_ST_
All	SPA	32	8.3	7.9	3	0.63	0.80	<0.001	∞ (20)	0.014
	IRE	26	9.4	9.3	4	0.67	0.80	<0.001	∞ (20)	0.014
	ITA	33	10.0	9.4	4	0.60	0.81	<0.001	∞ (20)	0.014
	GER	33	9.5	9.0	2	0.65	0.81	<0.001	∞ (20)	0.014
	CYP	32	10.4	9.9	8	0.62	0.82	<0.001	∞ (20)	0.013
	RUS	30	9.3	8.9	2	0.61	0.79	<0.001	∞ (20)	0.014
Males	SPA	16	7.3	6.8	5	0.61	0.79	<0.001	∞ (20)	0.018
	IRE	14	8.0	7.8	6	0.70	0.82	0.001	45.2 (20)	0.017
	ITA	16	7.9	7.5	2	0.62	0.80	<0.001	∞ (20)	0.014
	GER	16	8.3	7.9	4	0.68	0.83	<0.001	∞ (20)	0.017
	CYP	16	8.4	7.9	5	0.60	0.81	<0.001	∞ (20)	0.017
	RUS	14	6.6	6.5	1	0.59	0.77	<0.001	54.5 (20)	0.019
Females	SPA	16	7.1	6.6	1	0.66	0.80	0.001	53.2 (20)	0.022
	IRE	12	6.8	6.6	3	0.63	0.77	<0.001	∞ (20)	0.024
	ITA	17	8.1	7.1	3	0.58	0.81	<0.001	∞ (20)	0.022
	GER	17	7.6	6.8	1	0.62	0.79	<0.001	∞ (20)	0.022
	CYP	16	9.0	7.9	8	0.64	0.82	<0.001	∞ (20)	0.021
	RUS	16	8.4	7.3	4	0.62	0.79	<0.001	68.7 (20)	0.022

The genetic variability of the STR loci for each population was computed considering all individuals and male and female genotypes separately: *n*, sample size; *n*_a_, average number of alleles/locus; *A*_r_, allelic richness; *A*_u_, number of unique alleles; *H*_O_, observed heterozygosity; *H*_E_, expected heterozygosity; *P*_HWE_, probability value for the Hardy-Weinberg Equilibrium test; χ**^2^** test with relative degrees of freedom (*df*) (Fisher exact test, all loci). Departure from HWE was significant in all populations after Bonferroni correction (α = 0.05, α' = 0.05/60 = 0.0008).

**Figure 3:**
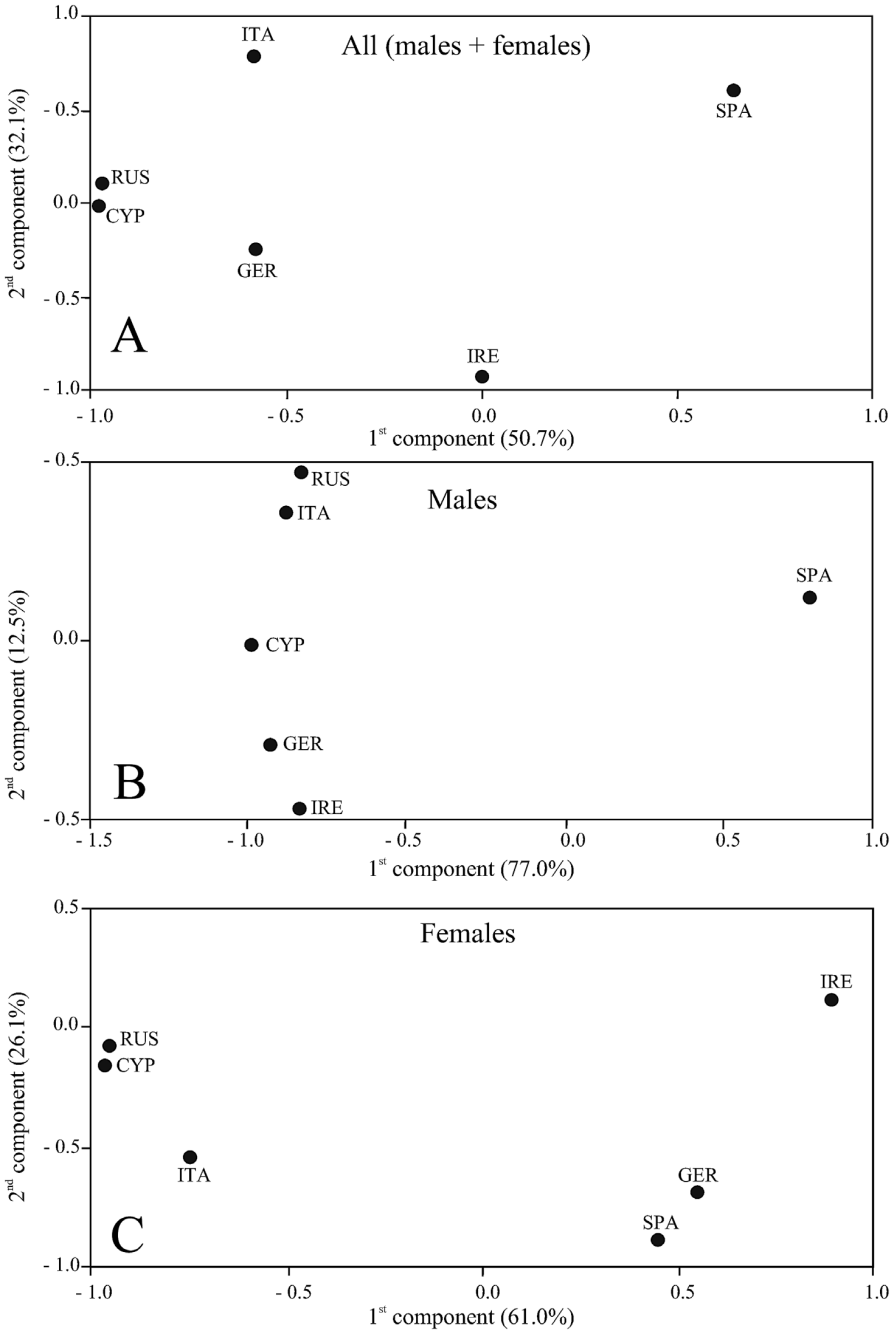
The Principal Component Analysis performed using the average pairwise *F*_ST_ distance values among STR genotypes. The percentage of total variance explained by each of the first two components is given. (A) All individuals. (B) Only males. (C) Only females.

We found that 98.6% of the total STR variability was partitioned within populations and 1.38% among them (*F*_ST_ = 0.014, *P*<0.001). In the PCA plot reported in Figure 3A, the first two components explained the 82.8% of the total variability, SPA being the most diverging population (e.g., *versus* CYP and RUS, *F*_st_ = 0.026 and 0.027, respectively: *P*<0.001). Non significant *F*_st_ distance values were found among ITA, GER, CYP and RUS (gene flow range: 3.87–6.07, Table 3).

**Table 3:** Estimates of population pairwise genetic distance values (*F*_ST_) and number of migrants (*N*_e_m).

		SPA	IRE	ITA	GER	CYP	RUS
All	SPA	-	3.24	4.05	3.01	4.09	2.96
	IRE	0.024**	-	4.23	3.43	3.31	2.80
	ITA	0.009	0.019*	-	5.43	4.37	6.07
	GER	0.017*	0.012*	0.008	-	4.58	3.87
	CYP	0.026**	0.016*	0.006	0.010	-	4.53
	RUS	0.027**	0.019*	0.005	0.010	0.002	-
Males	SPA	-	2.89	2.22	2.57	1.69	2.59
	IRE	0.037*	-	2.07	2.51	1.96	1.69
	ITA	0.022*	0.012	-	2.08	2.36	2.91
	GER	0.029*	0.001	0.001	-	3.03	2.09
	CYP	0.050**	0.012	0.008	0.004	-	4.09
	RUS	0.039*	0.020	0.004	0.012	0.012	-
Females	SPA	-	1.67	3.44	2.58	3.22	2.37
	IRE	0.021	-	1.79	2.18	3.05	2.75
	ITA	0.012	0.034*	-	3.13	3.38	2.76
	GER	0.009	0.027*	0.025*	-	3.71	3.77
	CYP	0.019	0.036*	0.008	0.029*	-	3.95
	RUS	0.031*	0.048**	0.015	0.028*	0.005	-

Estimates of population pairwise genetic distance (*F*_ST_) and number of migrants (*N*_e_m) were computed for all individuals, only males and only females. Below diagonal: *F*_ST_ pairwise distance values. Above diagonal: the effective number of migrants per generation (*N*_e_m); *, *P*<0.05; **, *P*<0.001; others, *P*>0.05.

#### Population genetic structure

**Figure 4:**
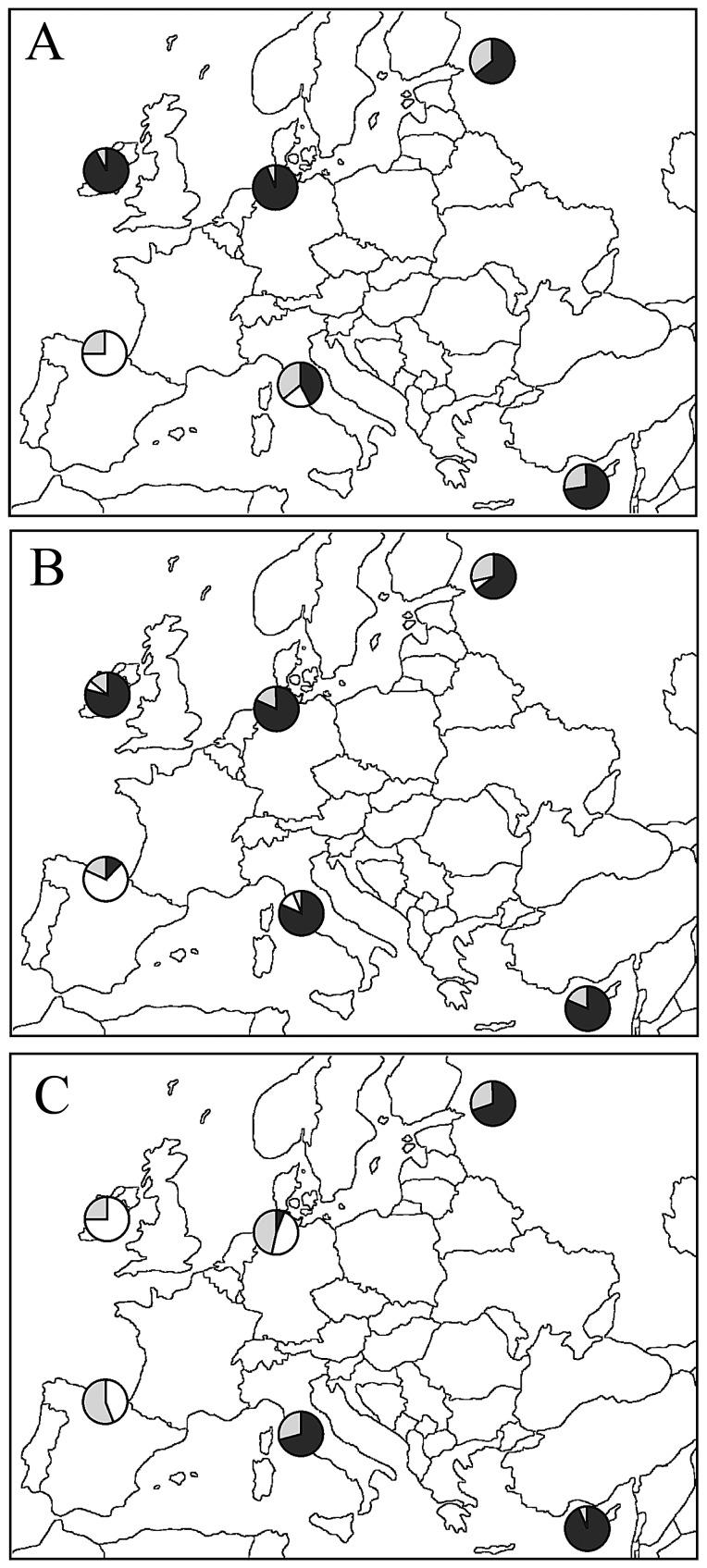
Bayesian admixture analysis as inferred using structure. The Δ*K* calculated according to Evanno *et al*. [61] was optimal for *K* = 2 in all computations. Each population was represented by a pie chart whose segments were proportional to the number of specimens assigned to cluster I (black), to cluster II (white) or which showed admixed genotypes (grey). Threshold value for assignment to each cluster was Q_i_ = 0.80 (Table S4). (A) All individuals. (B) Only males. (C) Only females.

In Figure 4A the whole sample size was taken into account. Bayesian clustering analysis indicated that barn swallows could be divided into two genetic groups (*K* = 2, see also Table S4). Genetic differentiation was strong between SPA and all other populations, and Spanish individuals were mostly assigned to the cluster II (Q_I_ = 0.16; Q_II_ = 0.84). While ITA showed the highest number of barn swallows with admixed genotype (*n = *12: Q_I_ = 0.59 and Q_II_ = 0.41, see Table S4), a slight differentiation was found between (IRE + GER) and (CYP + RUS) population pairs, yet their individuals were all assigned to the cluster I (Q_I_ range: 0.82–0.94). When we excluded from the Bayesian clustering analysis the STR loci showing null alleles and deviating from HWE (Hir4, Hir7, and Hir24: see above), barn swallows were assigned to two genetic groups and results matched those obtained when the entire set of loci was taken into account (data not shown).

### Microsatellite DNA: males *versus* females

#### Genetic variability

Average levels of *H*_O_ and *H*_E_ across all loci and populations were relatively homogeneous and showed deficiency of heterozygotes in both male and female barn swallows (Fisher exact test, *P*<0.001 all loci, Table 2). IRE and CYP showed the highest number of private alleles in males and females, respectively (Table 2). Females showed global *F*_ST_ values higher than males in all populations (on average, females: *F*_ST_ = 0.022, *P*<0.001; males: *F*_ST_ = 0.017, *P* = 0.003; Table 2). The majority of the total STR variability was partitioned within populations in both males and females (98.2% and 97.8%, respectively: data not shown). Differences between sexes were found in the PCA plot of average *F*_ST_ distance values. Males were highly differentiated along the 1^st^ component (77.0% of the STR diversity: Figure 3B), while in the females the 2^nd^ component explained a significant portion of the total variability (26.1%: Figure 3C). In males, SPA was the only divergent population with *F*_ST_ values ranging from 0.022 to 0.050 (*versus* ITA and CYP, respectively, *P*<0.05: Table 3). Significant *F*_ST_ distance values were disclosed among other pairs of populations in females but not in males (Table 3). In females, the largest genetic distance values were found between IRE and ITA (0.034), IRE and CYP (0.036) and IRE and RUS (0.048) (all *P*<0.05, Table 3), whereas no genetic differentiation was disclosed among ITA, CYP and RUS as well as among IRE, SPA and GER. Gene flow was, on average, higher in females than in males (average *N*_e_m: females  = 2.92, males  = 2.45, Table 3).

#### Population genetic structure

Bayesian clustering analysis performed in males and females separately suggested the occurrence of two groups (*K* = 2, Figure 4B, 4C; see also Table S4). For each population, males and females showed a largely different genetic make-up that could be inferred only partially when the entire sample size was included in the analysis (see above). When we used only the genotypic data inferred from the males, SPA diverged from all other populations and showed the highest membership value to the cluster II (Q_I_ = 0.21; Q_II_ = 0.79, Figure 4B). No similar evidence was observed in the other populations: in these latter, most of the individuals were assigned to the cluster I (Q_I_ range: 0.75–0.92) and only a very few were assigned to the cluster II or showed admixed genotype (Figure 4B, Table S4). When we used only the genotypic data inferred from the females, two different groups were disclosed. One included SPA, IRE and GER, which showed the highest assignment values to the cluster I (Q_I_ = 0.67, 0.84 and 0.70, respectively: Figure 4C). The other comprised ITA, CYP and RUS, which showed the highest assignment values to the cluster II (Q_II_ = 0.83, 0.89 and 0.78, respectively: Figure 4C, Table S4). When SPA was excluded from the Bayesian clustering analysis, the pattern showed by the females was very similar to that inferred using all populations (Figure S1 *versus* Figure 4C): in ITA, CYP and RUS the majority of the individuals were included in the black cluster, while IRE and GER included many birds assigned to the white cluster. On the contrary, when the SPA birds were excluded, a not negligible level of genetic diversity came to the fore among the males (Figure S1 *versus* Figure 4B), and pointed to the occurrence of three genetic groups (IRE + GER, ITA + CYP, and RUS), although the *F*_ST_ distance values were not significantly different among each other (not shown).

#### Dispersal

Males and females were tested for sex-biased dispersal using either all populations separately or keeping SPA on its own and grouping all of the other ones. In the first case, females showed higher inter-population *F*_IS_ and *vAI*_C_ values than males (females: *F*_IS_ = 0.225, *vAI*_C_ = 10.982; males: *F*_IS_ = 0.217, *vAI*_C_ = 8.829, Table 4). However, these differences were not statistically significant (all parameters). In the second case, *F*_ST_ (females: *F*_ST_ = 0.004; males: *F*_ST_ = 0.028, *P* = 0.015) and *R* values (females: *R* = 0.007; males: *R* = 0.045, *P* = 0.017) were significantly different and suggested female-biased dispersal. This finding was supported further by higher values of *F*_IS_ in the females than in the males as well as by the negative value of *mAI*_C_ in the females (*F*_IS_ = 0.234, *mAI*_C_ = −0.040); however, these differences were not statistically significant (all parameters, Table 4).

**Table 4:** Sex-biased dispersal tests.

	All populations			SPA *versus* all others		
	Males	Females	*P* -value	Males	Females	*P* -value
*F*_IS_	0.217	0.225	0.389	0.217	0.234	0.243
*F*_ST_	0.011	0.015	0.763	0.028	0.004	0.015*
*R*	0.017	0.025	0.752	0.045	0.007	0.017*
*mAI*_C_	−0.052	0.051	0.594	0.041	−0.040	0.444
*vAI*_C_	8.829	10.982	0.147	15.580	11.110	0.898

*F*_IS_, *F*_ST_, relatedness (*R*), mean assignment index (*mAI*_C_) and variance of mean assignment index (*vAI*_C_) were estimated on males and females considering either single populations or by separating Spanish barn swallows from all other ones. Significance (*P*) (two-tailed test) was assessed using the randomization method of [10]; *, *P*<0.05.

## Discussion

There are a few studies focusing on migratory bird species and relying on the use of molecular DNA markers (e.g., sandhill crane [63]; black-throated blue warbler [64]; reed warbler [65]). This likely occurs because species with elevated mobility are expected to show a higher level of gene flow and a weaker genetic structure than sedentary ones [66]–[68]). However, none of these studies accounted for sex in the genetic analysis. Only Ortego *et al.* [12] investigated the correspondence between population genetic structure and natal dispersal by analyzing males and females separately but in a non-migratory passerine (*Cyanistes caeruleus*, blue tit). On the contrary, studies focusing on other vertebrate species with sex-biased dispersal and taking into account the sex of individuals in the genetic analysis are known [16], [17], [69]. In the barn swallow, the use of either mitochondrial or microsatellite DNA pointed to the admixed genetic structure of the European populations investigated so far [23], [24], [26], and significant genetic (mtDNA) distances were found only among barn swallows of different continents [33]. Nevertheless, in this species, the genetic pattern of males and females has never been compared.

### Mitochondrial DNA

The mtDNA analysis did not suggest any significant structuring of the genetic variability (Figure 2), as it might be expected in a species with female-biased dispersal and male philopatry [69]. The star-like shape of the network, which included two ancestral haplotypes (H1 and H4), as well as the bell-shaped curve of the MD were consistent with a recent demographic expansion of the studied barn swallow populations (Figure 2, Table S2) [26], [33]. Such a scenario was statistically well supported (Fig. 2: *D* = −2.047, *F*_S_ = −56.028 and *R*_2_ = 0.040, all *P*<0.001), with the SSD statistic (*P* = 0.022) being an exception. However, although all tests we employed may be sensitive to unknown structure within populations, [70] stressed that the SSD statistic was actually the less powerful. Furthermore, when only the females were analyzed, a model of expansion was supported also by the SSD statistic (*P* = 0.063). Overall, we felt confident in considering that such a deviation from neutrality was very likely due to demographic changes rather than selective processes, as the McDonald-Kreitman test was not significant in the entire dataset [71]). This result is in agreement with the relatively recent barn swallow range and demographic expansion due to the proliferation of human settlements providing widespread availability of suitable nest sites [33].

The 11.6% of total mtDNA variability was partitioned among populations, a value much higher than that (<2%) found by Dor *et al*. [24], who analyzed populations of *H. r. rustica* and *H. r. transitiva* (Middle East). However, we feel confident that this result was due to the divergence of MED, the easternmost and northernmost population in our sampling scheme. Indeed, when MED was excluded from the analysis the mtDNA genetic structure promptly disappeared (Figure 1, Table S3).

### Microsatellite DNA: general overview

The loci of the microsatellite DNA investigated in this study showed a pattern of variability similar to that reported by [47], [48], with very high degree of polymorphism and low level of relatedness among the genotyped barn swallows (Table 1). Microsatellites did not show any evidence of Linkage Disequilibrium. However, a significant departure from HWE was found in all populations due to a deficiency of heterozygotes (Table 2). This result was possibly caused by either the occurrence of null alleles at a few STR loci or the Wahlund effect [72], which, in turn, pointed to sub-structuring due to sourcing from different populations with different allele frequencies [73]. As discussed by [63], who studied migratory sandhill cranes (*Grus canadensis*), a deficiency of heterozygotes may be common in migratory species when a given population consists of local and immigrant individuals with different origin. Overall, departures from HWE would seem also a deficit intrinsic to the barn swallow, which, however, do not necessarily affect the result of the genetic analysis. Indeed, Bayesian procedure does not require perfect equilibria to cluster individuals, yet it attempts to minimize such departures within groups [73]. Rodríguez-Ramilo *et al*. [74] evaluated the accuracy of some Bayesian clustering methods when both Hardy-Weinberg and Linkage Equilibria were not fully respected. They found that structure could reliably determine the correct number of clusters also for *F*_ST_ values as low as 0.01; hence, we did not exclude any STR locus from our analysis. Nevertheless, we also showed that the output of structure did not change when Hir4, Hir7 and Hir24 loci were ruled out (see Results).

### Microsatellite DNA: males + females, males only, females only

Santure *et al*. [23] and Dor *et al*. [24] investigated *H. rustica* including European populations with different morphology and migratory behaviour. Nevertheless, both mitochondrial and microsatellite DNA markers did not disclose any significant genetic structure. By contrasting an equal number of males and females from six localities across Europe, we show significantly more population structure in males than females. This difference in structure can be explained by dependence of dispersal on sex (Figure 3, 4; Tables 3, S4). Different from [23], in our study the pairwise *F*_ST_ computations as well as the PCA and the Bayesian clustering analysis revealed a genetic picture never before reported. While we cannot exclude that the discrepancy between the two studies could be due to the different distribution of the sampling sites, it should be noted that Santure *et al*. [23] used a set of STR markers (6 loci) smaller than that (10 loci) employed in the present study, and did not provide sex ratio of the investigated sample. Hence, it seems likely that our analysis has more power to detect any genetic structuring of populations than that performed by [23]. Overall, the PCA carried out by using the average pairwise *F*_ST_ distance values computed among all populations as well as the Bayesian clustering analysis (males + females), pointed to the strong divergence between SPA and all other populations (Figure 3A, 4A, Table 3). This result was even more evident when we used only the male genotypes (Figure 3B, 4B, Table 3). For instance, in the Bayesian analysis, most of the SPA individuals grouped together in the cluster II, whereas the males from all of the other populations were mainly assigned to the cluster I (Figure 4B). Balbontín *et al*. [27] obtained similar results in a field study. They investigated long-term trends in natal dispersal of northern (Denmark) and southern (Spain) Europe barn swallow populations. They found female-biased natal dispersal in both populations and male philopatry six times higher in the Spanish than in the Danish population. In our study, the genetic differentiation showed by the SPA population could be due to a particularly high rate of natal philopatry of males. We would like to stress that in the barn swallow the choice of the first breeding site is crucial, as it can determine where an individual will reproduce for the rest of its life. Balbontín *et al*. [27] suggested that the probability of philopatry, which is related to the fitness in terms of longevity, may depend on ecological factors related to the breeding site, although both the livestock farming and the architecture of rural buildings could also influence the choice between philopatry and dispersal [75]. Compared to the Spanish population, the lack of differentiation among all of the other ones could be due to the lower natal philopatry of their males. These populations, a mix of resident (philopatric) and immigrant (dispersing) individuals, showed reciprocal small genetic distances (Figure 3B, Table 3), and suggested that the barn swallow's dispersal behaviour should be regarded as a rather plastic trait [27]. However, when the SPA population was excluded from the Bayesian clustering analysis (only males), a not negligible degree of genetic differentiation was disclosed across Europe (Figure S1 *versus* Figure 4B). Although this result could seem related to the occurrence of differences in the migratory routes as we have suggested for the females (see next paragraph), we feel more confident in stating that male philopatry in each population is the primary cause for the population genetic structure we have found.

### Genetic structure and migratory behaviour (males *versus* females)

When only the females were taken into account the genetic scenario was strikingly different compared to that inferred using the males or the entire sample size. The average *F*_ST_ distance values (Table 3; with related PCA of Figure 3C) as well as the Bayesian clustering (Figure 4C) marked out two groups of females: one (central to western Europe) included SPA, IRE and GER, the other (central to eastern Europe) comprised ITA, CYP and RUS. The genetic distance between the two groups was significant (*F*_ST_ = 0.020, *P*<0.001, data not shown). Admixed genotypes were more frequent and gene flow level higher in the females than in the males (Figure 4, Tables 3, S4), this pointing to a higher dispersal rate and a lower philopatry of the first group compared to the second (female-biased dispersal) [4], [8], [9], [27].

When the clustering of the females was considered the occurrence of a significant latitudinal component could not be ruled out (e.g., compare SPA *versus* IRE and CYP *versus* RUS). This, in turn, suggested that gene flow among populations could be partially influenced by the axis of migration. However, the divergence between ITA and GER also suggests that dispersal does not strictly occur along a North-South axis. On the other hand, the genetic differentiation between eastern and western populations resembles what has been observed for other passerine species showing a clear migratory divide in central Europe [19], [76]. While clear evidences for the existence of a migratory divide do lack in the barn swallow, ringing data indicate that the autumn migratory routes of eastern and western populations of this species are different. Barn swallows from western Europe head for the Iberian Peninsula, while those of eastern populations travel down throughout the eastern Mediterranean and the Middle East. Again, barn swallows from central Europe may travel South straight across the Mediterranean or south-west to Spain [20]. This pattern of migration roughly parallels the results obtained by Ambrosini *et al*. [25] regarding the migratory connectivity in *H. rustica*. This analysis, which was carried out on a large dataset of ringing recoveries, produced weight for the existence of two main clusters: one includes birds breeding in south-west Europe and wintering in central Africa, the other comprises birds breeding in northern and eastern Europe and wintering in southern Africa. The genetic relationships disclosed in the present study, on one side, between RUS and CYP and, on the other, between SPA, GER and IRE, fit to the groups described by [25] and to the main migratory movements summarized in [20]. Furthermore, mark-recapture data provided by BirdLife Cyprus (A.C. Author pers. comm. 2012) also point to a single migration route between RUS and CYP. Whereas according to Ambrosini *et al*. [25] ITA should be separated from the RUS and CYP populations, our results point to the occurrence of some genetic kinship among the females of these countries (Figure 4A, Table S4). Overall, this may either indicate that the pattern of gene flow is only partially related to the migratory movement or suggest a poorly documented migratory route that connects central Italy, north-west Russia and Cyprus. Actually, the geographic area of birds recovered in Italy or ringed in Italy and recovered abroad is huge and include several eastern and south-eastern countries (e.g., Turkey and Greece, [77]). These wide connections, probably due to the position of the Italian Peninsula in the Mediterranean, should be further investigated by extending the number of the Italian sampling locations in order to attempt to disentangle a very complex pattern of gene flow.

When only the males were considered, the very high philopatry of the Spanish population forced the whole genetic scenario by isolating the latter against all of the other ones (see previous paragraph). On the contrary, when the SPA males were excluded, we disclosed slight differences among the studied populations that pointed to the occurrence of three groups: (IRE + GER), (ITA + CYP) and RUS (Figure S1). This pattern showed some degree of correspondence with the female clustering, thus suggesting that the mechanisms driving the direction of dispersal movements are probably the same in both sexes. Nevertheless, being aware that further investigations are needed, we suggest do not over-interpreting these results.

### Sex-biased dispersal

Cases of inconsistency between population structures inferred from mtDNA or from STR variability are well known. A greater population differentiation can be detected using nuclear rather than mitochondrial markers (e.g.: [78], [79], this study) but the opposite may occur as well (e.g.: [71], [80]). Although both natal and breeding dispersal may enable high connectivity among populations [1], [2], [5], [6], breeding dispersal is virtually absent in the barn swallow [27]. Hence, sex-biased natal dispersal represents the best explanation fitting the observed discordance, although tests for the whole dataset failed to obtain significance for all parameters (Table 4). However, as stressed by [10], their overall outcome must be discussed in light of the amount of combined factors that may affect the statistical power, namely the dispersal rate and the bias intensity, the sampling design and the number of investigated STR loci [16], [81]. For instance, when the dispersal rate and the sex-bias are not strong *vAI*_C_ seems the most powerful test, followed by *mAI*_C_ and *F*_ST_, which work better with less than 20 loci [10]. In this study, *vAI*_C_ was higher in the females than in the males, thus pointing to the occurrence of female-biased dispersal. When the tests were performed and SPA was separated from all other populations, *R* and *F*_ST_ values were significantly lower in the females than in the males, *mAI*_C_ value was negative (Table 4) and *F*_IS_ value was lower in males than in females, thus suggesting high philopatry for the Spanish males.

In conclusion, we partially confirmed published data reporting the lack of a significant partition of diversity using mtDNA markers, while, in contrast to previous studies, we detected significant genetic structure by using nuclear DNA markers. The different scenarios observed between sexes could be explained with the non-random patterns of gene flow likely mediated by female natal dispersal and significant variability in male philopatry among barn swallow populations. Our results emphasize the importance of taking into account the sex of sampled individuals to obtain unbiased results on species showing a different pattern of dispersal between males and females.

## Supporting Information

Figure S1 **Bayesian admixture analysis as inferred using structure performed excluding the SPA population in male and female barn swallows.** Δ*K* was optimal for *K* = 2, all computations. Each population was represented by a pie chart whose segments were proportional to the number of individuals assigned to cluster I (black), cluster II (white) or which showed admixed genotypes (grey). Threshold value for assignment to each cluster was Q_i_ = 0.80.
(DOC)

Table S1 **The sample size (** ***n*** **= 186) of this study and the mtDNA sequences downloaded from the GenBank (** ***n*** **= 16).** Population (Pop), country, region, locality (latitude/longitude, Lat/Long), type of tissue, sample size for STR/mtDNA analysis, and the number of ND2 mtDNA haplotypes are given. The number of male (M) and female (F) individuals genotyped with mitochondrial and STR markers was given (unavailable on line for MED population). GenBank accession codes for KRD and MED populations are reported in [33].
(DOC)

Table S2 **Estimates of mtDNA genetic diversity (averagez± SD) as computed for each population and for the whole dataset.** Sites: number of segregating sites.
(DOC)

Table S3 Average mtDNA pairwise distance values (Phi_ST_) as computed among all populations; *, ***P*** <0.05; **, ***P*** **<0.001; others,** ***P*** **>0.05.**
(DOC)

Table S4 **Proportion of estimated membership of all populations to each cluster (Q_I_ and Q_II_) as inferred by structure with** ***K*** **= 2 (** **Figure 4** **).** Computations were performed using: (i) all individuals (*n* = 186), (ii) males (*n* = 92) and (iii) females (*n* = 94). The number of specimens assigned to the cluster I (*n*_I_) and II (*n*_II_) was reported as well as the number of individuals showing admixed genotype (*n*_mix_). The threshold value for admixed assignment was Q_i_ = 0.80.
(DOC)
