# Supplementary material for: Large-Scale Patterns of Genetic Variation in a Female-Biased Dispersing Passerine: The Importance of Sex-Based Analyses
Source: PLoS One. 2014 Jun 2;9(6):e98574. doi: 10.1371/journal.pone.0098574 (PMC4041750; doi:10.1371/journal.pone.0098574)
Supplement: Figure S1 — Bayesian admixture analysis as inferred using structure performed excluding the SPA population in male and female barn swallows. ΔK was optimal for K = 2, all computations. Each population was represented by a pie chart whose segments were proportional to the number of individuals assigned to cluster I (black), cluster II (white) or which showed admixed genotypes (grey). Threshold value for assignment to each cluster was Qi = 0.80. (DOC) [file pone.0098574.s001.doc]

**Supporting Information**

| 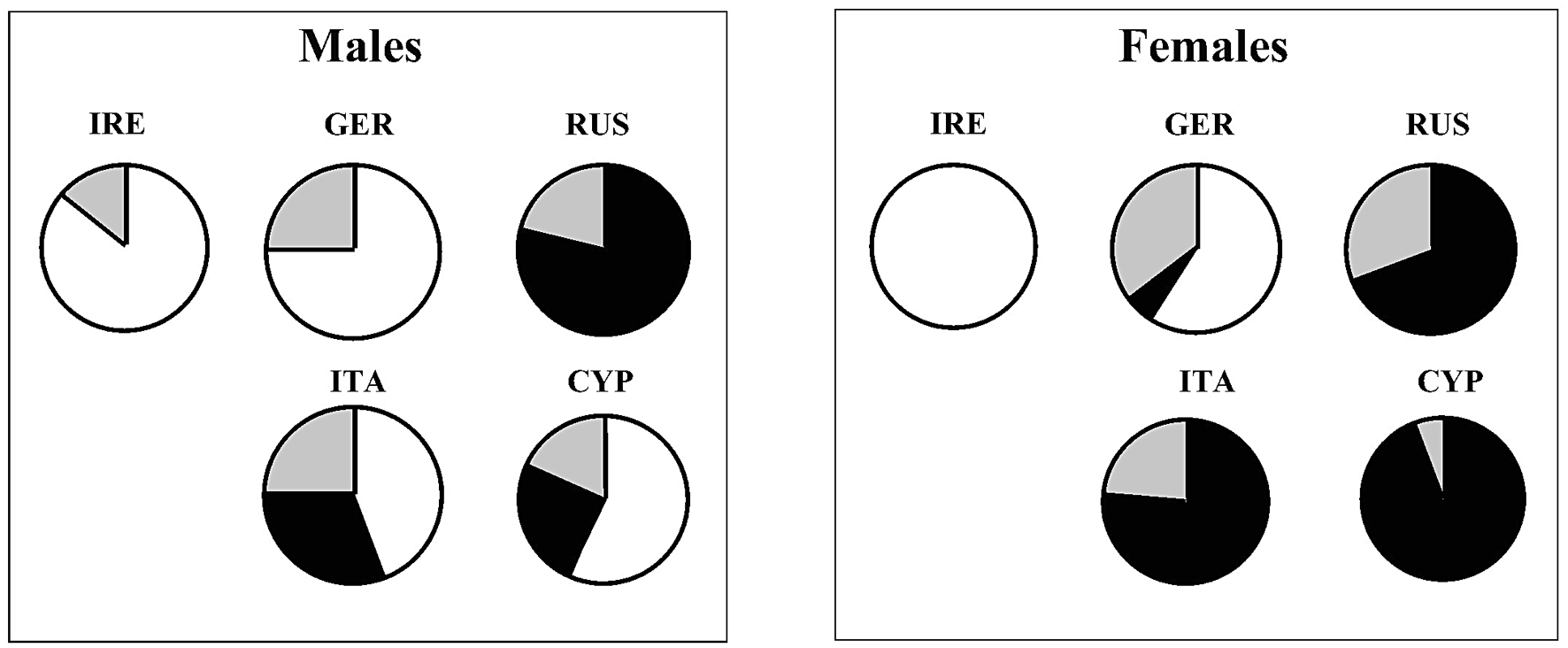 |
| --- |

**Figure S1**. Bayesian admixture analysis as inferred using structure performed excluding the SPA population in male and female barn swallows. ∆*K* was optimal for *K* = 2, all computations. Each population was represented by a pie chart whose segments were proportional to the number of individuals assigned to cluster I (black), cluster II (white) or which showed admixed genotypes (grey). Threshold value for assignment to each cluster was Q_i_ = 0.80.
