# Supplementary material for: Large-Scale Patterns of Genetic Variation in a Female-Biased Dispersing Passerine: The Importance of Sex-Based Analyses
Source: PLoS One. 2014 Jun 2;9(6):e98574. doi: 10.1371/journal.pone.0098574 (PMC4041750; doi:10.1371/journal.pone.0098574)
Supplement: Table S1 — The sample size ( n = 186) of this study and the mtDNA sequences downloaded from the GenBank ( n = 16). Population (Pop), country, region, locality (latitude/longitude, Lat/Long), type of tissue, sample size for STR/mtDNA analysis, and the number of ND2 mtDNA haplotypes are given. The number of male (M) and female (F) individuals genotyped with mitochondrial and STR markers was given (unavailable on line for MED population). GenBank accession codes for KRD and MED populations are reported in [33]. (DOC) [file pone.0098574.s002.doc]

**Supporting Information**

| Pop | Country | Locality | Lat/Long | Tissue | STR  (*n*) | mtDNA  (*n*) | Haplotype number |
| --- | --- | --- | --- | --- | --- | --- | --- |
| SPA | Spain | Gorliz | 43°24′ N; 02°57′ W | Blood | 32 | 10 | 1, 4, 8, 31 |
| IRE | Ireland | Tullynisk | 53°07’ N; 07°54′ W | Blood | 26 | 10 | 1, 4, 23, 30 |
| ITA | Italy | Orti-Bottagone | 42°57′ N; 10°35′ E | Blood | 33 | 10 | 1, 4, 5, 10, 14, 23 |
| GER | Germany | Itzehoe | 53°56′ N; 09°31′ E | Blood | 33 | 10 | 1, 4, 12, 13, 23, 25, 26, 27 |
| CYP | Cyprus | Polis | 35°02′ N; 32°25′ E | Blood | 32 | 10 | 1, 2, 3, 4, 6, 13, 20, 21, 29 |
| RUS | Russia | Lake Ladoga | 60°40′ N; 32°56′ E | Blood | 30 | 10 | 1, 4, 11, 13, 16, 23 |
| KRD | Russia | Krasnodar | 45°22′ N; 39°26′ E | - | - | 7 | 1, 4, 7, 9, 28 |
| MED | Russia | Medvedevo | 60°59′ N; 38°33′ E | - | - | 9 | 4, 15, 17, 18, 19, 22, 24 |

**Table S1**. The sample size (*n* = 186) of this study and the mtDNA sequences downloaded from the GenBank (*n* = 16). Population (Pop), country, region, locality (latitude/longitude, Lat/Long), type of tissue, sample size for STR/mtDNA analysis, and the number of ND2 mtDNA haplotypes are given. GenBank accession codes for KRD and MED populations are reported in [33].
