# Supplementary material for: Large-Scale Patterns of Genetic Variation in a Female-Biased Dispersing Passerine: The Importance of Sex-Based Analyses
Source: PLoS One. 2014 Jun 2;9(6):e98574. doi: 10.1371/journal.pone.0098574 (PMC4041750; doi:10.1371/journal.pone.0098574)
Supplement: Table S2 — Estimates of mtDNA genetic diversity (averagez± SD) as computed for each population and for the whole dataset. Sites: number of segregating sites. (DOC) [file pone.0098574.s003.doc]

**Supporting Information**

|  | Sample | Haplotypes | Sites | Haplotype diversity | Number of pairwise differences | Nucleotide diversity |
| --- | --- | --- | --- | --- | --- | --- |
|  | (*n*) | (*n*) | (*n*) | (*h* ± SD) | (*k* ± SD) | (π ± SD, %) |
| SPA | 10 | 4 | 3 | 0.71 ± 0.11 | 0.93 ± 0.69 | 0.09 ± 0.07 |
| IRE | 10 | 4 | 3 | 0.73 ± 0.10 | 0.93 ± 0.69 | 0.09 ± 0.07 |
| ITA | 10 | 6 | 6 | 0.84 ± 0.10 | 1.36 ± 0.91 | 0.13 ± 0.09 |
| GER | 10 | 8 | 7 | 0.96 ± 0.06 | 2.20 ± 1.32 | 0.21 ± 0.14 |
| CYP | 10 | 9 | 8 | 0.97 ± 0.05 | 2.02 ± 1.24 | 0.19 ± 0.13 |
| RUS | 10 | 6 | 5 | 0.91 ± 0.06 | 1.47 ± 0.97 | 0.14 ± 0.10 |
| KRD | 7 | 5 | 5 | 0.86 ± 0.14 | 1.62 ± 1.08 | 0.16 ± 0.12 |
| MED | 9 | 7 | 8 | 0.94 ± 0.07 | 1.94 ± 1.21 | 0.19 ± 0.13 |
| Total | 76 | 31 | 27 | 0.88 ± 0.03 | 1.73 ± 1.02 | 0.17 ± 0.11 |

**Table S2.** Estimates of mtDNA genetic diversity (average ± standard deviation, SD) as computed for each population and for the whole dataset. Sites: number of segregating sites.
