# Supplementary material for: Large-Scale Patterns of Genetic Variation in a Female-Biased Dispersing Passerine: The Importance of Sex-Based Analyses
Source: PLoS One. 2014 Jun 2;9(6):e98574. doi: 10.1371/journal.pone.0098574 (PMC4041750; doi:10.1371/journal.pone.0098574)
Supplement: Table S3 — Average mtDNA pairwise distance values (PhiST) as computed among all populations; *, P <0.05; **, P <0.001; others, P >0.05. (DOC) [file pone.0098574.s004.doc]

**Supporting Information**

|  | SPA | IRE | ITA | GER | CYP | RUS | KRD | MED |
| --- | --- | --- | --- | --- | --- | --- | --- | --- |
| SPA | - | - | - | - | - | - | - | - |
| IRE | - 0.061 | - | - | - | - | - | - | - |
| ITA | - 0.004 | - 0.022 | - | - | - | - | - | - |
| GER | 0.056 | 0.021 | 0.012 | - | - | - | - | - |
| CYP | - 0.012 | - 0.012 | - 0.017 | 0.040 | - | - | - | - |
| RUS | 0.091* | 0.062 | - 0.008 | 0.035 | 0.041 | - | - | - |
| KRD | - 0.027 | - 0.027 | - 0.027 | 0.031 | - 0.041 | 0.038 | - | - |
| MED | 0.378** | 0.372** | 0.312** | 0.289** | 0.277** | 0.292** | 0.303** | - |

**Table S3**. Average mtDNA pairwise distance values (Phi_ST_) as computed among all populations; *, *P* < 0.05; **, *P* < 0.001; others, *P* > 0.05
