# Supplementary material for: Large-Scale Patterns of Genetic Variation in a Female-Biased Dispersing Passerine: The Importance of Sex-Based Analyses
Source: PLoS One. 2014 Jun 2;9(6):e98574. doi: 10.1371/journal.pone.0098574 (PMC4041750; doi:10.1371/journal.pone.0098574)
Supplement: Table S4 — Proportion of estimated membership of all populations to each cluster (QI and QII) as inferred by structure with K = 2 ( Figure 4 ). Computations were performed using: (i) all individuals (n = 186), (ii) males (n = 92) and (iii) females (n = 94). The number of specimens assigned to the cluster I (n I) and II (n II) was reported as well as the number of individuals showing admixed genotype (n mix). The threshold value for admixed assignment was Qi = 0.80. (DOC) [file pone.0098574.s005.doc]

**Supporting Information**

|  | All | | | | | Males | | | | | Females | | | | |
| --- | --- | --- | --- | --- | --- | --- | --- | --- | --- | --- | --- | --- | --- | --- | --- |
|  | Q_I_ | Q_II_ | *n* _I_ | *n* _II_ | *n* _mix_ | Q_I_ | Q_II_ | *n*_I_ | *n*_II_ | *n*_mix_ | Q_I_ | Q_II_ | *n*_I_ | *n*_II_ | *n*_mix_ |
| SPA | 0.16 | 0.84 | 0 | 24 | 8 | 0.21 | 0.79 | 2 | 11 | 3 | 0.67 | 0.33 | 0 | 7 | 9 |
| IRE | 0.94 | 0.06 | 24 | 0 | 2 | 0.84 | 0.16 | 11 | 1 | 2 | 0.84 | 0.16 | 0 | 9 | 3 |
| ITA | 0.59 | 0.41 | 14 | 7 | 12 | 0.81 | 0.19 | 13 | 2 | 1 | 0.17 | 0.83 | 12 | 0 | 5 |
| GER | 0.94 | 0.06 | 31 | 0 | 2 | 0.92 | 0.08 | 13 | 0 | 3 | 0.70 | 0.30 | 1 | 8 | 8 |
| CYP | 0.85 | 0.15 | 23 | 0 | 9 | 0.92 | 0.08 | 13 | 0 | 3 | 0.11 | 0.89 | 15 | 0 | 1 |
| RUS | 0.82 | 0.18 | 19 | 0 | 11 | 0.75 | 0.25 | 9 | 1 | 4 | 0.22 | 0.78 | 11 | 0 | 5 |

**Table S4.** Proportion of estimated membership of all populations to each cluster (Q_I_ and Q_II_) as inferred by structure with *K* = 2 (Figure 4). Computations were performed considering (i) all individuals (*n* = 186), (ii) males (*n* = 92) and (iii) females (*n* = 94). The number of specimens assigned to the cluster I (*n*_I_) and II (*n*_II_) was reported as well as the number of individuals showing admixed genotype (*n*_mix_). The threshold value for admixed assignment was Q_i_ = 0.80.
